# Strong and Durable Wood Designed by Cell Wall Bulking Combined with Cell Lumen Filling

**DOI:** 10.3390/polym16010093

**Published:** 2023-12-28

**Authors:** Yaoyao Dong, Yanran Qi, Xiaoying Dong, Yongfeng Li

**Affiliations:** 1 State Forestry and Grassland Administration Key Laboratory of Silviculture in Downstream Areas of the Yellow River, College of Forestry, Shandong Agricultural University, Taian 271018, China; sdaudyy@126.com (Y.D.); qyran1994@163.com (Y.Q.); 2 Key Laboratory of Bio-Based Material Science and Technology of Ministry of Education, Northeast Forestry University, No. 26 Hexing Road, Harbin 150040, China

**Keywords:** Maleic anhydride, GMA-EGDMA monomer, wood–polymer composite, mechanical properties, dimensional stability, ecofriendly

## Abstract

Traditional wood–polymer composite (WPC) based on the in situ polymerization of ethylene unsaturated monomers in the cellular cavity of wood is significant for the high-value-added utilization of low-quality wood. However, this type of WPC has the problems of volatile monomers, low conversion rates, odor residue, and poor compatibility between the polymer and wood interface, which hinder its promotion and application. In this study, a two-step process of cell wall bulking in combination with cell lumen filling was prepared to modify wood using Maleic anhydride (MAN) as the bulking agent and GMA-EGDMA (molar ratio 2:1) as the active monomer system. The results indicate that the modulus of rupture (MOR) (125.19 ± 8.41 MPa), compressive strength (116.38 ± 7.69 MPa), impact toughness (55.4 ± 2.95 KJ m^−2^), and hardness (6187 ± 273 N) of the bulking–filling wood composite materials were improved by 54%, 56%, 36%, and 66%, respectively, compared with those of poplar wood. These properties were superior to those of the traditional styrene (PSt)-WPC and even exceeded the performance of *Xylosma congesta* (Lour.) Merr, a high-quality wood from northeast China. Meanwhile, the mass loss of wood composite materials with bulking–filling treatment was only 2.35 ± 0.05%, and the internal structure remained intact, presenting excellent decay resistance. Additionally, the treatment also significantly improved the thermal and dimensional stability of the wood composites. This study provides a theoretical basis and guidance for realizing the high-value-added application of low-quality wood and the preparation of highly durable wood-based composites.

## 1. Introduction

The impact of global warming and environmental degradation has led to a worldwide consensus on the development and use of low-carbon, environmentally friendly materials to address climate change. Wood, as a green and renewable resource, offers advantages such as sustainability, aesthetic value, and a high strength-to-weight ratio to help global green and low-carbon development [1]. Currently, due to the scarcity of high-quality timber resources, fast-growing tree species such as poplar have become crucial plantation species because of their advantages of fast growth, wide distribution, and strong adaptability. However, poplar wood, despite its fast growth, is flimsy, low-strength, easy to decay and deform, and difficult to utilize, which significantly limits its application and development [2]. In order to fully utilize wood resources and prepare environmentally friendly materials to alleviate the contradiction between supply and demand, it is necessary to modify fast-growing poplar [3].

In recent years, the technology for improving wood properties has developed and gained attention, particularly in the area of polymer-reinforced wood. A wood–polymer composite (WPC) is produced by immersing unsaturated monomers into wood under vacuum pressure and triggering a free radical polymerization reaction of the monomers by heating [4], radiation or ultrasonic waves [5], and electron beam irradiation [6]. It has good dimensional stability, hardness, strength, and abrasion resistance, making it widely used in the fields of construction, furniture, musical instruments, and industrial equipment [7]. For instance, previous studies have utilized various monomers, including styrene [8], methyl methacrylate [9], n-butyl acrylate [10], alkoxysilane coupling agents [11], vinyl monomers [12], methyl methacrylate (MMA) [13], and unsaturated polyester resins [14], to impregnate the wood and polymerize, filling the cell lumina to enhance the strength and dimensional stability of the wood. Research has shown that the composite treatment of wood with functional monomers, such as propylene oxide and methyl methacrylate monomers [15], ethylene/methyl methacrylate, and styrene/acrylonitrile monomers [16], can improve the comprehensive performance of wood. Additionally, research is being conducted on the interaction between copolymers and wood to better understand the mechanism of their action [17]. Furthermore, studies have investigated methods to enhance the cost-effectiveness of WPC production processes [7]. However, problems such as monomer volatility and interfacial gaps between polymers and wood continue to restrict the improved properties and practical applications of WPC [18].

**Figure 1:**
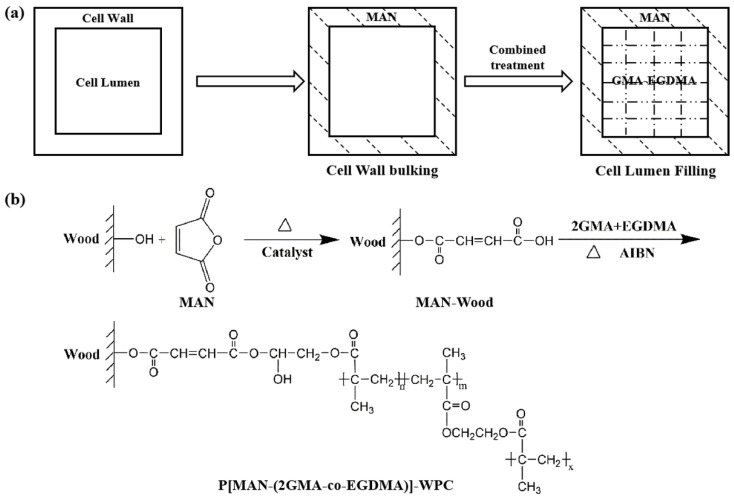
Schematic diagram of preparation and structure of composites: (**a**) schematic diagram of preparation and (**b**) reaction principle.

Therefore, this study proposed a new strategy of cell wall bulking combined with cell lumen filling. Maleic anhydride (MAN), a small organic molecule that can penetrate the bulk cell wall, was selected to bulk the cell wall and participate in the bonding reaction of the wood hydroxyl group with the functional group of MAN to reduce the intracell wall pores and water-absorbing groups inside the bulk cell wall. Then, the bifunctional monomers glycidyl methacrylate (GMA) and ethylene dimethacrylate (EGDMA) were used for polymerization in situ in the cell lumina of wood. This process filled the pores of the cell lumen and bonded to the cell wall, eliminating the composite interface between the polymer and the wood. As a result, the coupling improvement of the bulking–filling composite materials improved the comprehensive performance of the wood (Figure 1a).

## 2. Materials and Methods

### 2.1. Materials

The poplar specimens (*Populus ussuriensis*) were harvested from the Experimental Forestry Farm in Hao’er Mountain, Heilongjiang. The samples were from poplar wood with a DBH of 25–30 cm. The average water content was 10.4% (mean value of 5 samples), and the average density was 0.33 ± 0.03 g cm^−3^ (mean value of 5 samples).

Maleic anhydride (MAN, Shanghai Chemical Reagent Factory), acetone (Tianjin Kaitong Reagent Co., Ltd., Tianjin, China), glycidyl methacrylate (GMA, Shanghai Yuanji Chemical Co., Ltd., Shanghai, China), ethylene glycol methacrylate (EDGMA), and azodiisobutyronitrile (AIBN, Shanghai No. 4 Reagent Factory, Shanghai, China) were analytical pure and used directly.

### 2.2. Preparation of MAN-Wood

MAN was dissolved in acetone to a concentration of 20%, and the proper pyrimidine was added as a catalyst. After stirring, the poplar wood specimen was immersed into the solution under 0.08 MPa for 20 min and 0.8 MPa for 20 min successively. After the pressure removal, the sample was wrapped in tin foil and stood at room temperature for 24 h, and then the wrapped poplar wood specimen was heated for 8 h under the condition of 110 °C. Finally, the poplar wood specimen was vacuum-treated (0.08 MPa) at room temperature for 30 min to remove residual acetone odor. The resulting wood was labeled MAN-Wood.

### 2.3. Preparation of P[MAN-(2GMA-co-EGDMA)]-WPC

GMA and EGDMA were co-mixed according to the molar ratio of 2:1. The initiator AIBN, which accounted for 1% of the mass of the monomer solution, was dissolved in the above monomer mixed solution to form a GMA-EGDMA bifunctional monomer. Then, the MAN-Wood was impregnated with the bifunctional monomer according to the method in 2.2 to construct bulking–filling wood composite materials. The schematic diagram of the reaction is shown in Figure 1b.

### 2.4. Characterization

A scanning electron microscope (SEM, QUANTA2000, FEI, Hillsboro, NH, USA) was utilized to observe the microscopic morphology of the wood. Before the analysis, the samples were sprayed with gold for 15 s using a 10 mA current. The chemical composition of the specimens was examined by Fourier transform infrared spectroscopy (FTIR, Magna IR560, Nicolet, Madison, WI, USA); the resolution ratio was 4 cm, and 40 spectra were accumulated. The crystallization of the samples was determined by X-ray diffraction (XRD, D/MAX2200VPC, Rigaku, Tokyo, Japan). XRD tests were performed at 40 kV and 30 mA. Patterns were collected over the 2θ range of 5–60° and a scanning speed of 5 °C min^−1^. A thermogravimetric analyzer (TGA, Q500, Waters, Taunton, MA, USA) was used to determine the thermal stability of the specimens. Samples were heated from 35 to 600 °C at a heating rate of 10 °C min^−1^ under a flow of nitrogen atmosphere (100 mL min^−1^). A Contact Angle Tester (JC2000A, Powereach, Shanghai, China) was used to determine the water contact angle of the wood. All instantaneous contact angles used a water droplet of 5 μL volume. The droplet was placed at three positions on each sample. The instantaneous contact angles were determined by the average of the measured values.

### 2.5. Properties Evaluation

The physical and mechanical properties of the specimens were tested according to the national standard GB/T 1927.2-2021 [19]. The sample size of modulus of rupture (MOR) was 20 mm × 20 mm × 300 mm (radial (R) × tangential (T) × longitudinal (L)). The sample size of the determination of ultimate stress in compression parallel to grain was 20 mm × 20 mm × 30 mm (R × T × L); the sample size of impact toughness was 20 mm × 20 mm × 300 mm (R × T × L); and the sample size of hardness was 50 mm × 50 mm × 20 mm (R × T × L). Five specimens were used for each test. The mechanical properties of untreated and treated samples were tested using a universal testing machine (AG-10TA, Shimadzu Corporation, Japan, Kyoto). For hardness testing, the indenter (a steel ball with a diameter of 11.3 mm) connected to the loading plate of the testing machine was pressed to the surface of the specimen. A preload of 1~2 N was applied to stabilize the sample. Loading and then increasing by 1000 cows in 15 s was the goal of the load, while keeping the force for 25 s. The actual contact area under the indentation was used to calculate the hardness of the specimen. The load deformation data were obtained at a sampling rate of 10 data points per second. The sample size of antiswelling efficiency (ASE) was 20 mm × 20 mm × 20 mm (R × T × L). The samples were immersed in distilled water for 720 h. The dimensions of each sample were measured after different soaking times. The average data of 5 samples for each measurement are given. 

The dimensional stability was evaluated according to the antiswelling rate (*ASE*) and water absorption reduction rate (*RWA*). *ASE* is defined as follows:

(1) $$ASE\;\left(\%\right)=\frac{{VSE}_{u}-{VSE}_{t}}{{VSE}_{u}}\times 100$$

where *VSE_t_* and *VSE_u_* are the volume swelling efficiency of treated and untreated wood, respectively. 

The volume swelling (*VSE*) efficiency is:

(2) $$VSE\;\left(\%\right)=\frac{V_{1}-V_{0}}{V_{0}}\times 100$$

where *V*_1_ is the sample volume after immersion in water for 720 h, and *V*_0_ is the sample volume before immersion.

The *RWA* (reduction in water absorptivity) is calculated as follows:

(3) $$RWA\;(\%)=100\times ({WA}_{i}-{WA}_{0})/{WA}_{0}$$

where *WA_i_* and *WA*_0_ are the water absorptivity of treated and untreated wood, respectively. The *WA* is defined as follows:

(4) $$WA\;(\%)=100\times (W_{i}-W_{0})/W_{0}$$

where *W_i_* and *W*_0_ are the samples’ weights after and before immersion, respectively.

The wood preservation (Corrosion resistance) test was carried out using the Chinese forestry industry standard LYT1283-2011 [20], and the test strain is *Gloeophyllum trabeum* (Pers. Ex Fr.) Murr. Five samples were required for each performance test. The test samples were autoclaved for 30 min and placed in an incubator with a relative humidity of 83% and a temperature of 28 °C. After 12 weeks of fungal decay, the reduction in mass of the woodchip specimen was weighed as mass loss.

The sample size of weight percent gain (*WPG*) was 20 mm × 20 mm × 20 mm (R × T × L). The impregnation of specimens under different treatment conditions was measured and calculated by weighing method and the *WPG* can be calculated as the following equation:

(5) $$WPG=\frac{W_{1}-W_{0}}{W_{0}}\times 100\%$$

where *W*_0_ and *W*_1_ are the oven-dried weight of the control and impregnated wood. There were 6 duplicate specimens in each group.

## 3. Results

### 3.1. Cell Wall Bulking

**Figure 2:**
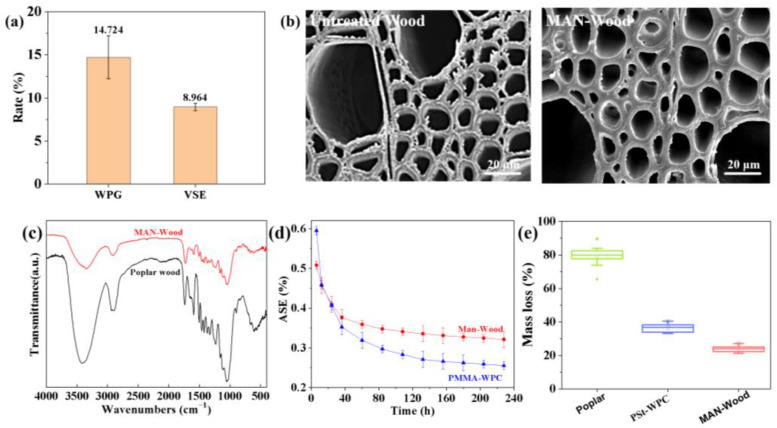
Characterization of MAN-Wood: (**a**) bulking results, (**b**) SEM images, (**c**) FTIR spectra, (**d**) ASE, (**e**) corrosion resistance.

Figure 2a shows the degree of cell wall bulking of wood measured by WPG and VSE. After treatment with MAN, the average WPG of poplar specimens was 14.72 ± 2.50%, and the average VSE was 8.96 ± 0.43%. These results suggest that MAN had a significant bulking effect on the wood [21]. The cross-section of MAN-Wood showed a porous lumen structure, similar to the SEM image of untreated poplar, suggesting that MAN may have infiltrated into the cell wall (Figure 2b).

The intensity of the O–H bond in MAN-Wood at 3373 cm^−1^ was weaker than that of the untreated material, indicating a reduction in the amount of –OH groups in MAN-Wood. This suggests that the original –OH groups in the cell wall had a nucleophilic substitution reaction with the cyclic anhydride bond of MAN, resulting in the elimination of the hydroxyl groups. Compared with the fundamental frequency of the O–H spectral band of poplar wood (3395 cm^−1^), the spectral band of MAN-Wood was shifted to a lower frequency. This shift indicates that the –OH did not originate entirely from the wood, and part of it came from the carboxyl group on the polymer. In other words, MAN reacted with the hydroxyl group of the wood to produce the carboxyl group. The intensity of the carbonyl band at 1733 cm^−1^ was significantly intensified compared with the untreated wood, indicating the involvement of MAN in the chemical reaction. The C=C telescopic vibration peak at 1635 cm^−1^ further proved that MAN was grafted onto the wood cell wall. The spectra showed that MAN was grafted into the cell wall of the wood through the C(=O) –O stretching vibration at 1240 cm^−1^, the C–O–C asymmetric stretching vibration at 1164 cm^−1^ [22], and the C–O symmetric stretching vibration at 1055 cm^−1^ (Figure 2c). Therefore, the anhydride molecules grafted to the wood cell wall, indicating a chemical binding between MAN and wood cell wall components.

Figure 2d shows the ASE change curves of MAN-Wood at different times. After 228 h of water immersion, the ASE of MAN-Wood reached 32.1 ± 2.11%, which is significantly higher than the ASE of plasticized wood (25.5 ± 1.17%) prepared by using the cell wall expander MAN, coupling agent GMA, and monomer MMA. The low conversion rate of the system as a whole is attributed to the high volatility of the monomer MMA [23]. According to the corrosion resistance results of MAN-Wood (Figure 2e), it can be observed that the untreated poplar experienced a mass loss of 79.28 ± 6.45%. In contrast, MAN-Wood experienced a mass loss that was 55.33 ± 2.05% lower than that of the untreated poplar and significantly higher than that of the traditional polystyrene (PSt)-WPC (36.53 ± 2.51%) [24]. These results can be attributed to the fact that MAN fills the cell wall of the wood and chemically eliminates a large number of water-absorbing hydroxyl groups that absorb water. The treatment destroys the living environment of brown rot fungi within the cell wall and improves the preservative ability of MAN-treated wood.

### 3.2. Preparation and Characterization of Bulking–Filling Wood Composite Materials

P[MAN-(2GMA-co-EGDMA)]-WPC is a bulking–filling wood composite material made by impregnating a bifunctional monomer consisting of GMA and EGDMA (in a molar ratio of 2:1) into MAN-wood, followed by heat-initiated polymerization. The reaction conditions for the polymerization process are detailed in Table 1. The effects of reaction temperature and time on ASE (24 h) were analyzed by ANOVA (Table 2), and both factors were found to have significant effects on ASE at the selected levels. The optimal reaction conditions were found to be 110 °C and 8 h, indicating favorable conditions for the ring cleavage of the epoxy group of GMA under the influence of MAN.

**Figure 3:**
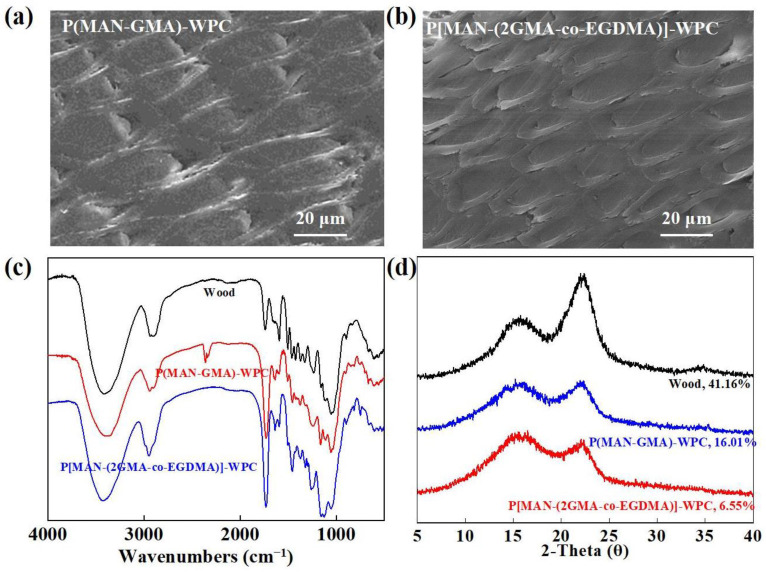
Characterization and chemical analysis of P[MAN-(2GMA-co-EGDMA)]-WPC: (**a**,**b**) SEM images, (**c**) FTIR spectra, (**d**) XRD patterns.

P[MAN-(2GMA-co-EGDMA)]-WPC was produced under optimal reaction conditions. For comparison, P(MAN-GMA)-WPC was obtained by impregnating GMA monomer into MAN-Wood under the same conditions. The SEM results are shown in Figure 3a, and Figure 3b shows that GMA homopolymerized in the MAN-Wood voids and uniformly filled the cell lumens of the wood to form P(MAN-GMA)-WPC. This structure differs from that of poplar wood material and MAN-Wood (Figure 2b). The results show that GMA was tightly bound to the cell walls of MAN-Wood, indicating strong interfacial interactions. This may be attributed to the bonding of GMA to the cell walls of MAN-Wood via the epoxy groups to nucleophilic substitution reactions with hydroxyl or carboxyl groups on MAN-Wood. Similarly, the polymer in P[MAN-(2GMA-co-EGDMA)]-WPC was well bound to the wood-matrix-phase interface and filled the cell lumen, suggesting that GMA played a crucial role in interfacial compatibility.

Compared with untreated wood, the FTIR patterns of P(MAN-GMA)-WPC and P[MAN-(2GMA-co-EGDMA)]-WPC showed a decrease in intensity in the O–H band at 3412 cm^−1^, with a tendency to move to the higher frequencies, which was mainly attributed to the reaction between the polymers and hydroxyl groups of the wood, resulting in a decrease in the hydroxyl content (Figure 3c). Meanwhile, the intensity of the carbonyl band at 1732 cm^−1^ increased sequentially, indicating that GMA and EGDMA were grafted onto the wood. The C–O stretching vibration at 1234 cm^−1^, the C–O–C asymmetric stretching vibration at 1163 cm^−1^, and the C–O symmetric stretching vibration at 1055 cm^−1^ were enhanced compared with those of the untreated poplar, which also proved that the filling of GMA and EGDMA. These results are in consistent with the expected reaction principle (Figure 1b).

The relative crystallinity of P[MANMan-(2GMA-co-EGDMA)]-WPC and P(MANMan-GMA)-WPC was calculated using the Segal method (Equation (6)) [25]. The results show that CRI_P[MANMan-(2GMA-co-EGDMA)]-WPC_ was 6.55% and CRI_P(MANMan-GMA)-WPC_ was 16.01%. The relative crystallinity of the composites decreased significantly after the bulking–filling treatment decreased dramatically compared with the untreated wood. The increase in the proportion of amorphous components of the wood is attributed to the reaction of MAN-Wood with GMA, which resulted in the polymer linking onto the wood cell walls. Furthermore, the involvement of EGDMA in the polymerization reaction of GMA in the wood cell lumen caused the relative crystallinity of P[MAN-(2GMA-co-EGDMA)]-WPC (6.55%) to be even lower than that of P(MAN-GMA)-WPC (16.01%) (Figure 3d).

C_r_I = (I_002_ − I_am_)/I_002_ × 100% (6)

In the equation: C_r_I—percentage of relative crystallinity;

I_002_—(002) lattice diffraction angle of the maximum intensity (any unit);

I_am_—represents the scattering intensity of the amorphous background when the 2θ angle is close to 18°, which is the same as the I_002_ unit.

### 3.3. Mechanical Properties of Bulking–Filling Wood Composite Materials

**Figure 4:**
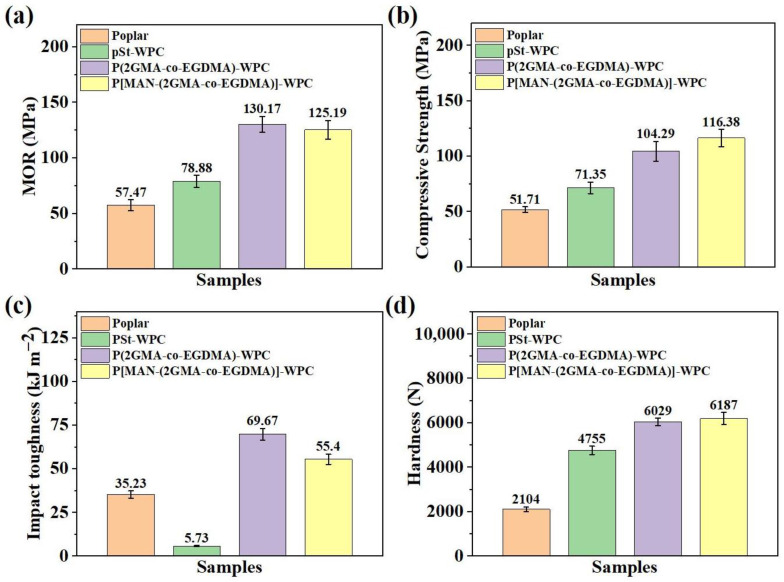
Mechanical properties of P[MAN-(2GMA-co-EGDMA)]-WPC: (**a**) MOR, (**b**) compressive strength, (**c**) impact toughness, (**d**) hardness.

To further evaluate the mechanical properties of the bulking–filling wood composite materials P[MAN-(2GMA-co-EGDMA)-WPC], poplar wood material, traditional PSt-WPC, and filling-treated wood composite material P(2GMA-co-EGDMA)-WPC were selected as controls. The MOR of P[MAN-(2GMA-co-EGDMA)]-WPC reached 125.19 ± 8.41 MPa, which was significantly higher than that of untreated wood (57.47 ± 4.74 MPa), and PSt-WPC (78.88 ± 5.33 MPa) was comparable to that of P(2GMA-co-EGDMA)-WPC (Figure 4a). The compressive strength parallel to the grain of P[MAN-(2GMA-co-EGDMA)]-WPC was 116.38 ± 7.69 MPa, which was 55.6% and 38.7% higher than that of the untreated poplar and PSt-WPC, respectively. It was also higher than that of P(2GMA-co-EGDMA)-WPC (Figure 4b). Although the impact toughness of P[MAN-(2GMA-co-EGDMA)]-WPC (55.40 ± 2.95 KJ m^−2^) was slightly lower than that of P(2GMA-co-EGDMA)-WPC, it was significantly better than that of the untreated poplar (35.23 ± 2.06 KJ m^−2^) and PSt-WPC (5.73 ± 0.34 KJ m^−2^) (Figure 4c). The treatment of the wood cell wall with bulking–filling resulted in a tighter bond between the molecular chains of the wood components. The mechanical properties of P[MAN-(2GMA-co-EGDMA)]-WPC were improved compared with untreated poplar and PSt-WPC and were comparable to those of P(2GMA-co-EGDMA)-WPC. The impact toughness of P[MAN-(2GMA-co-EGDMA)]-WPC was slightly lower than that of the filling-treated wood, which may be attributed to the degradation of the wood components by the carboxyl groups generated when MAN treated the wood. It is worth noting that the bulking–filling treatment resulted in the mechanical properties of the modified poplar wood exceeding those of the high-quality wood from northeast China [24].

### 3.4. Durability and Dimensional Stability of Bulking–Filling Wood Composite Materials

**Figure 5:**
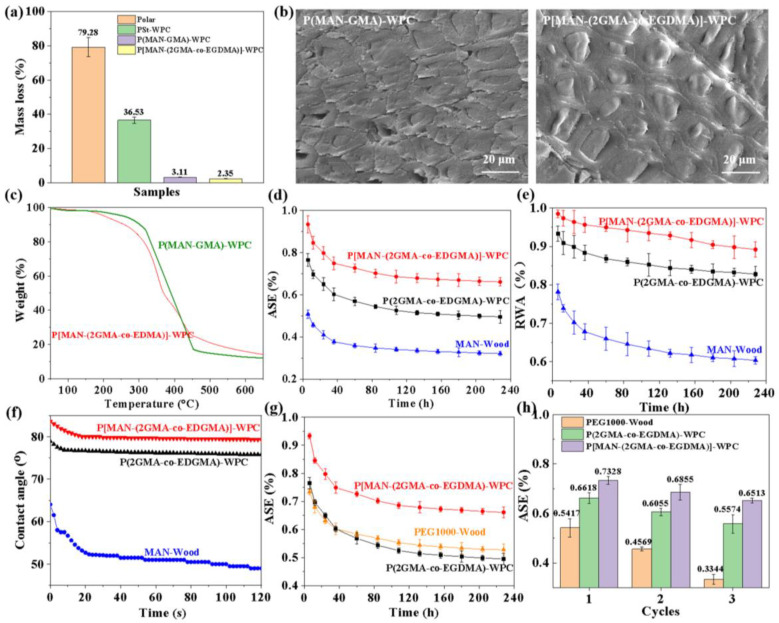
Durability and dimensional stability of P[MAN-(2GMA-co-EGDMA)]-WPC: (**a**) corrosion resistance, (**b**) SEM images, (**c**) TG curve, (**d**) ASE, (**e**) RWA, (**f**) contact angle, (**g**) contrast of ASE, (**h**) ASE under three cycles of immersion.

Further evaluation of the durability of P[MAN-(2GMA-co-EGDMA)]-WPC revealed a mass loss of only 2.35 ± 0.05% when corroded by brown-rot fungi. This is significantly lower than the weight loss of untreated poplar (79.28 ± 5.66%) and the traditional PSt-WPC (36.53 ± 1.92%) (Figure 5a). The cross-section of the bulking–filling wood composite material remained structurally intact after decay. The polymer was tightly bonded to the wood cell wall, and the cell walls were not significantly decayed by brown-rot fungi (Figure 5b), which is consistent with the mass loss rate. The bulking–filling treatment may have prevented the penetration of microorganisms into the wood cell walls, thus providing excellent decay resistance to the modified wood. In the rapid degradation stage, the thermogravimetric (TG) of P(MAN-GMA)-WPC and P[MAN-(2GMA-co-EGDMA)]-WPC were higher than that of untreated poplar, indicating improved thermal stability of the modified wood (Figure 5c). The addition of MAN increased the interfacial interaction between the polymer and the wood matrix phase, crosslinking the polymer in the cell lumen and increasing the density of crosslinking sites and cohesion. Thus, the bulking–filling system improved the thermal stability of the wood to some extent.

Figure 5d shows that the ASE of the treated wood decreased throughout the water immersion cycle of 228 h. Additionally, the ASE of P[MAN-(2GMA-co-EGDMA)]-WPC was higher at 66.1 ± 2.37% compared with P(2GMA-co-EDGMA)-WPC at 49.5 ± 3.14%. Figure 5e further demonstrates that the bulking–filling wood composite material had a water absorption resistance (RWA) of 89.2 ± 2.38% after 228 h, which was significantly higher than that of MAN-Wood at 60.3 ± 1.56% and P(2GMA-co-EDGMA)-WPC at 82.8 ± 2.44%. During the process of bulking and bonding the wood cell wall, MAN occupies the microcapillaries and eliminates some of the hydroxyl groups. Additionally, the polymerization of bifunctional monomer in the cell lumen eliminates some of the hydroxyl groups and occupies the pores of the cell lumen, which prevents water from penetrating into the interior of the wood. The water contact angle on the tangential section of the modified wood was significantly higher than that of the MAN-Wood and decreased slowly (Figure 5f). This suggests that the filling treatment reduced the hydrophilicity of the wood. The carbonyl and carboxyl groups produced by the partial ring-opening reaction of MAN-Wood reduced the water contact angle of the wood surface.

The dimensional stability of the bulking–filling wood composite materials was evaluated by comparing P(2GMA-co-EDGMA)-WPC with polyethylene glycol (PEG) 1000-treated wood. The ASE of P[MAN-(2GMA-co-EGDMA)]-WPC decreased from 93.4 ± 1.77% to 66.07 ± 2.31%, which was significantly higher than that of PEG1000-Wood (52.8 ± 2.36%) and P(2GMA-co-EDGMA)-WPC (49.5 ± 2.64%) (Figure 5g) [23]. Thus, the bulking–filling treatment combines the advantages of both bulking and filling treatments, leading to the optimal ASE. To test the dimensional stability of these composites in more demanding environments, such as bathrooms and outdoor areas, a hot water (60 °C, 12 h)–cold water (25 °C, 12 h) cycle was used. The ASE of the wood-based composites was tested, and we found that the ASE of P[MAN-(2GMA-co-EDGMA)]-WPC decreased from 73.3 ± 1.56% to 65.1 ± 1%, which was higher than that of PEG1000-Wood (33.4 ± 1.98%) and P[MAN-(2GMA-co-EDGMA)]-WPC (55.7 ± 3.77%) (Figure 5h). PEG primarily blocks, restricts, and impedes the penetration of water molecules into the wood cell wall by occupying the pores. However, PEG does not form a chemical bond with the cell wall, causing it to dissolve easily after repeated hot–cold water immersion, which results in a decrease in dimensional stability. The bulking–filling treatment eliminated a large number of hydroxyl groups in the wood cell wall through a chemical reaction and achieved the filling of the polymer within the wood matrix. This reduces the loss of polymer and improves the dimensional stability of the wood substantially and permanently. This method is effective in improving the dimensional stability.

## 4. Conclusions

This study utilized MAN as the bulking agent and GMA-EGDMA (molar ratio 2:1) as the active monomer to prepare wood composite materials through a two-step method of bulking combined with a filling treatment. These monomers have high boiling points, which effectively overcome the bottleneck of traditional ethylene monomers that are prone to volatility and residual odor. The mechanical properties of low-quality poplar wood were improved by 36% to 66% through the combined bulking–filling treatment, respectively. The weight loss of the bulking–filling composite materials inoculated with brown-rot fungi was reduced by 77% compared with poplar wood, while maintaining the internal structure intact. This ultimately achieved the goal of enhancing its overall performance. This study analyzed the thermal and dimensional stability of low-quality poplar wood. These findings provide scientific guidance for designing novel wood composite materials to increase the added value of poplar wood.

## Figures and Tables

**Table 1:** Preparation of experimental design parameters and data sheets of P[MAN-(2GMA-co-EGDMA)]-WPC.

Number	Temperature (°C)	Time (h)	ASE (%)
1	80	2	58.92 ± 3.56
2	90	4	65.40 ± 5.28
3	100	6	70.22 ± 1.77
4	110	8	78.10 ± 3.21
5	90	6	66.98 ± 2.64
6	80	8	64.33 ± 4.53
7	110	2	67.07 ± 2.33
8	100	4	68.46 ± 3.37
9	100	8	73.55 ± 4.26
10	110	6	76.44 ± 1.75
11	80	4	60.35 ± 0.98
12	90	2	62.65 ± 1.54
13	110	4	71.92 ± 2.09
14	100	2	64.07 ± 1.17
15	90	8	68.57 ± 2.82
16	80	6	61.73 ± 4.97

**Table 2:** Preparation process parameters analysis of variance table.

	DEVSQ	Degree Freedom	Mean Square	F	Significance
Temperature	0.0446	3	0.0149	29.80	**
Time	0.0546	3	0.0182	36.40	**
Error	0.0046	9	0.0005		
Sum	10.38%	15			

F_0.05_ (3,9) = 3.86, F_0.01_ (3,9) = 6.99, ** highly significant.

## Data Availability

The data presented in this study are available on request from the corresponding author.
